# Interventions for improving clinical outcomes and health-related quality-of-life for people living with skeletal dysplasias: an evidence gap map

**DOI:** 10.1007/s11136-023-03431-z

**Published:** 2023-06-09

**Authors:** Naomi Moy, Darren Flynn, Josefa Henriquez, Luke B. Connelly, Luke Vale, Francesco Paolucci

**Affiliations:** 1 Department of Sociology and Business Law, University of Bologna, Bologna, Italy; 2 Department of Midwifery, Nursing and Health, Faculty of Health and Life Sciences, Northumbria University, Newcastle upon Tyne, UK; 3 Centre for the Business and Economics of Health, The University of Queensland, Brisbane, Australia; 4 Health Economics Group, Population Health Sciences Institute, Faculty of Medical Sciences, Newcastle University, Newcastle upon Tyne, UK; 5 Newcastle Business School, Faculty of Business and Law, University of Newcastle, Callaghan, Australia

**Keywords:** Skeletal dysplasia, Evidence gap map, Clinical outcomes, Quality-of-life, Psychosocial functioning

## Abstract

**Purpose:**

Skeletal dysplasias are rare genetic disorders that are characterized by abnormal development of bone and cartilage. There are multiple medical and non-medical treatments for specific symptoms of skeletal dysplasias e.g. pain, as well as corrective surgical procedures to improve physical functioning. The aim of this paper was to develop an evidence-gap map of treatment options for skeletal dysplasias, and their impact on patient outcomes.

**Methods:**

We conducted an evidence-gap map to identify the available evidence on the impact of treatment options on people with skeletal dysplasias on clinical outcomes (such as increase in height), and dimensions of health-related quality of life. A structured search strategy was applied to five databases. Two reviewers independently assessed articles for inclusion in two stages: titles and abstracts (stage 1), and full text of studies retained at stage 2.

**Results:**

58 studies fulfilled our inclusion criteria. The included studies covered 12 types of skeletal dysplasia that are non-lethal with severe limb deformities that could result in significant pain and numerous orthopaedic interventions. Most studies reported on the effect of surgical interventions (*n* = 40, 69%), followed by the effect of treatments on dimensions of health quality-of-life (*n* = 4, 6.8%) and psychosocial functioning (*n* = 8, 13.8%).

**Conclusion:**

Most studies reported on clinical outcomes from surgery for people living with Achondroplasia. Consequently, there are gaps in the literature on the full range of treatment options (including no active treatment), outcomes and the lived experience of people living with other skeletal dysplasias. More research is warranted to examine the impact of treatments on health-related quality-of-life of people living with skeletal dysplasias, including their relatives to enable them to make preference- and valued based decisions about treatment.

**Supplementary Information:**

The online version contains supplementary material available at 10.1007/s11136-023-03431-z.

## Background

Skeletal dysplasia is an overarching term for rare genetic disorders that are characterized by the abnormal development of bone and cartilage, which can also affect muscle, tendons, and ligaments. There are over 461 heterogeneous skeletal dysplasias [1] with an estimated overall prevalence of 2.3 per 10,000 live births [2]. The majority of skeletal dysplasias first present at a young age [3, 4].

There are numerous sub-types of skeletal dysplasias that are non-lethal, but can result in severe limb deformities and significant pain and discomfort that could lead to loss of mobility and require numerous orthopaedic interventions. One such sub-type are metaphyseal chondrodysplasias, which are a diverse group of genetic disorders characterized by flaring and irregularity of various metaphyses (long bones) and vertebrae (spinal bones) [3]. The most common metaphyseal chondrodysplasia is Metaphyseal Chondrodysplasia type Schmid (MCDS, ORPHA174). MCDS is an ultra-rare genetic condition, with an estimated prevalence of three to six individuals per million of the population [5]. It is caused by a mutation in the X-type collagen, with signal characteristics of short stature with abnormally short arms and legs (short-limbed dwarfism) and bowed legs (*genu varum*) [6]. A secondary sub-type are epiphyseal dysplasias which are grouped by the condition’s spinal involvement [7].

Understanding the lived experience of those with a skeletal dysplasia is important to providing optimal care throughout their healthcare journey [8]. Due to the nature of the conditions, those with skeletal dysplasia have increased levels of pain, and in adults with skeletal dysplasia those with higher levels of pain are more likely to report mental health concerns [9]. In addition to higher levels of pain, those with skeletal dysplasia have poorer health-related quality of life (HRQoL) outcomes and experience increased limitations to their physical functioning [10, 11].

The treatment and management of symptoms of those with a skeletal dysplasia is complex and unique to the individual, and is focused on improving symptoms, physical function and prevention of complications in later life. Treatments include non-medical and medical treatments, as well as corrective surgeries to improve deformities, physical function and reduce pain [6]. These interventions often commence from an early age, with surgical interventions occurring at different stages in an individual’s growth development and remaining growth potential [4]. Surgical treatment for symptoms of skeletal dysplasia continues to develop in an effort to reduce the number of repeated corrective surgical procedures [4]. Non-medical treatment includes orthoses and physical therapy, which can help correct particular deformities of the hip and knee, and posture [12]. Physical therapy may involve strengthening exercises for the lower extremities which, in part, may help with pain management. Further pain management options include the long-term use of analgesic medication and/or hot, moist packs [13].

With advances in genetic medicine, new treatments are emerging in the form of targeted drug therapies. These treatments have the potential to decelerate or prevent the development of skeletal dysplasias [14]. The efficacy of drug treatments varies by genetic condition: for example, growth hormone therapy is indicated as a treatment for short stature for people with achondroplasia or hypochondroplasia, but not MCDS [15]. In the case of MCDS, carbamazepine, a drug treatment for epilepsy and other seizure disorders, trigeminal neuralgia, and bipolar disorder is undergoing a clinical trial to establish its effectiveness for reducing endoplasmic reticulum stress and so improve bone growth and patient outcomes [6, 16].

Despite the treatment options available and the potential for emerging therapies, outcomes vary considerably between individuals affected by skeletal dysplasia. There are currently no systematic reviews or evidence gap maps of treatment options for skeletal dysplasias and their impact on clinical outcomes and HRQoL.

We formulated the following review question to produce an evidence gap map:

For children/adults diagnosed with non-lethal skeletal dysplasias with severe limb deformities and significant pain and discomfort that could lead to loss of mobility and require numerous orthopaedic interventions, what is the evidence for the impact of available treatment options on clinical outcomes and health-related quality of life?

## Methods

We adhered to the Preferred Reporting Items for Systematic Review and Meta-Analyses extension for Scoping Reviews (PRISMA-ScR) guidelines [17], and guidance for producing evidence gap maps [18].

### Eligibility criteria

#### Study designs

All study designs (qualitative and quantitative) with a sample size of ≥ 10 participants were eligible for inclusion in the review.

#### Population

Children or adults with a diagnosis of one of 30 skeletal dysplasias that shared similarities in terms of being non-lethal with severe limb deformities and significant pain and discomfort that could lead to loss of mobility and require numerous orthopaedic interventions. The list of conditions eligible for inclusion was developed by consulting with a geneticist with specialist expertise in skeletal dysplasias, specifically MCDS (see online resource 1). Studies were excluded if most participants (≥ 50%) did not have a diagnosis of these skeletal dysplasias.

#### Interventions

Any type of surgery, medical (pharmacological) or non-medical treatment: physical therapy, orthoses, supported self-management for health and lifestyle change, counselling, or specialist services for mental health.

#### Outcomes

Clinical outcomes (e.g., changes in height or skeletal measurements, correction of deformities, complication rate), and dimensions of HRQoL (physical functioning, pain, mental health/well-being, psychosocial functioning and satisfaction).

### Search strategy

The search strings to identify relevant studies were applied to the following bibliographic databases: MedLine, PsychInfo, CINHAL, EMBASE, and SCOPUS. The structured search strategy was devised using a combination of subject indexing terms and free text search terms covering the period up to March 2021. The search terms were identified through background literature screening. In addition, experts in the field of skeletal dysplasia were contacted to request papers that may not yet be available in the public domain. There was no restriction placed on publication date, but studies were restricted to English, Spanish or Italian publications. An example of the search strategy applied to EMBASE can be found in online resource – online resource 2.

### Selection of studies

Three reviewers (NM, JH, DF) independently screened the titles and abstracts identified by the search strategy. Studies retained at this stage were read in full-text, and their relevance for inclusion was independently determined by two reviewers (NM, JH) with reference to a study selection form (online resource 1). A third reviewer (DF) adjudicated on any disagreements between these two reviewers that could not be resolved via discussion.

### Risk of bias

Due to the extreme rarity of the genetic conditions in the general population, study designs were expected to be predominantly observational, with small sample sizes. For this reason, we did not assess methodological quality, as the primary aim was to develop an evidence-gap map.

### Data synthesis

A systematic mapping exercise was conducted to produce an evidence gap map of research across a range of treatment options and outcomes for people living with non-lethal skeletal dysplasias that are non-lethal with severe limb deformities and significant pain and discomfort that could lead to loss of mobility and require numerous orthopaedic interventions. A structured data extraction form was used to extract data to produce an evidence gap-map of: type of skeletal dysplasia; treatment type (surgical, pharmacological, physical therapy, supported self-management for health and lifestyle change, orthoses); outcome category (clinical, HRQoL [scales that report across dimensions of HRQoL—psychosocial functioning, mental health/wellbeing, pain, physical functioning, and satisfaction], or report on dimensions of HRQoL as single measure derived from HRQoL measurement scales, or single item measure scales (e.g. VAS for pain severity); and direction of treatment effect (whether the treatment had no effect, a positive, or negative impact on outcomes). Clustered bar graphs were produced to convey frequency of studies for: type of skeletal dysplasia and type of treatment; and type of treatment and type of outcomes. An evidence gap map was produced to convey the effects of treatments, by type, on outcomes for each type of skeletal dysplasia.

## Results

**Fig. 1:**
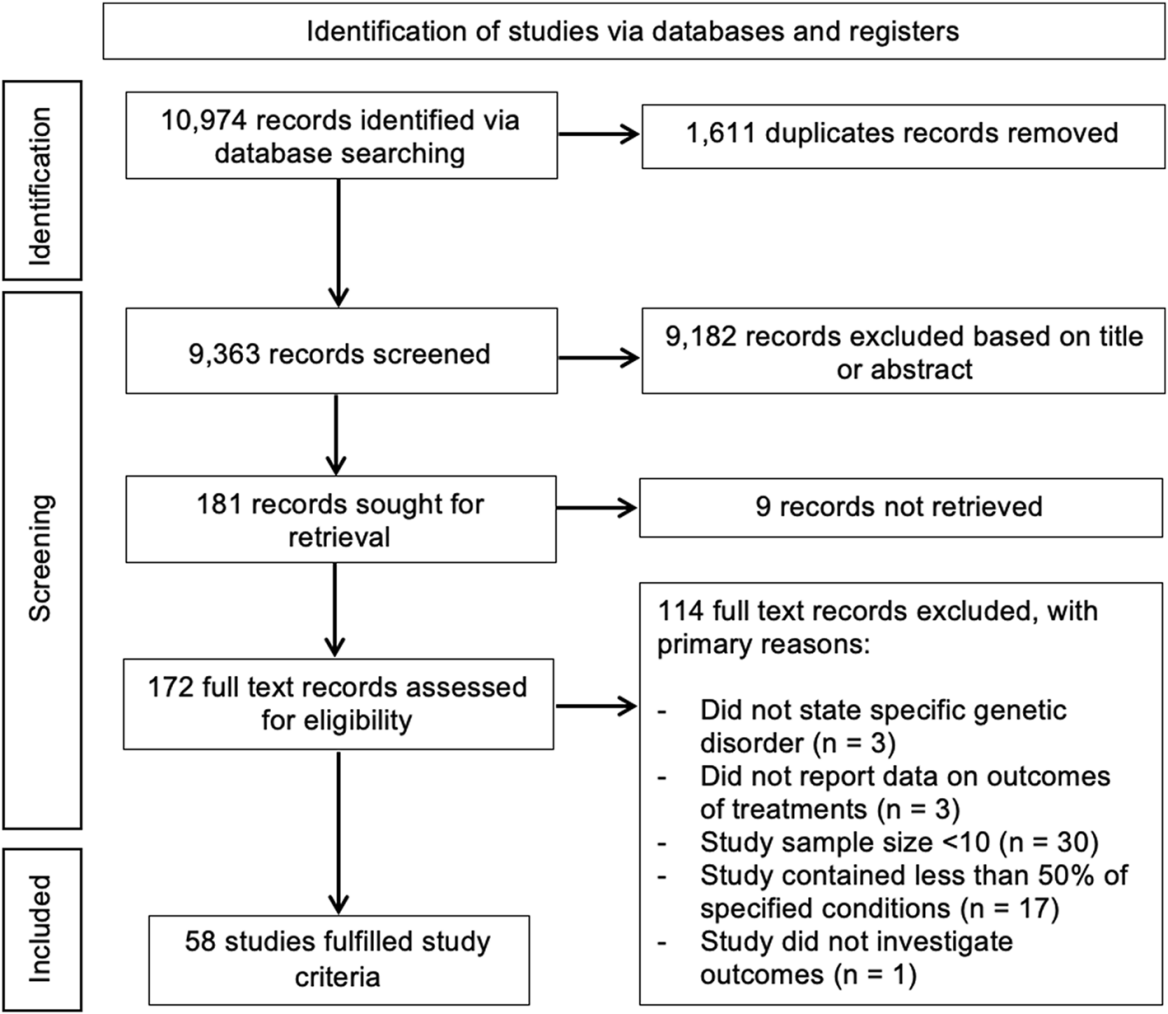
Flow diagram summarising the process used to identify the included studies. An overview of the characteristics of each included study is provided in online resource 3

A total of 10,974 records (9,363 after de-duplication) were identified by the search strategy, with 181 full text studies evaluated for inclusion in the evidence-gap map (Fig. 1). From these 181 studies, a total of 58 studies met the eligibility criteria [5, 15, 19–74].

Out of the 58 included studies, four were randomised control trials [22, 32, 53, 54], 25 were retrospective case studies [5, 23–25, 27, 28, 30, 34, 35, 39, 41, 42, 45, 46, 48, 49, 57, 59, 65–69, 71, 73], 15 were retrospective cohort studies [20, 21, 26, 29, 31, 36, 38, 40, 47, 55, 58, 60, 61, 70, 74], eight were controlled before and after studies [15, 51, 52, 56, 62–64, 72], two were prognostic studies [19, 37], and one study was a non-randomised control trial [50]. There were three cross-sectional studies that used patient focused surveys [33, 43, 44].

Included studies were conducted across 23 countries: Japan (*n* = 13) [15, 31, 34, 38–40, 43, 46, 53, 54, 63, 64, 72] United States of America (USA; *n* = 12) [19, 23, 25, 26, 28, 44, 55–58, 70, 71]; South Korea (*n* = 10) [35–37, 42, 48, 49, 59–61, 68]; United Kingdom (UK; *n* = 5) [22, 27, 30, 45, 51]; Italy (*n* = 4) [20, 21, 67, 74]; France (*n* = 2) [50, 66]; China (*n* = 2) [29, 41]; the Netherlands (*n* = 2) [65, 69]. One study was conducted each in Greece (62), Spain (47), and Turkey (24). Five of the studies utilised multi-national cohorts: Australia, Canada and UK (33); Australia, France, UK and USA (52); Croatia and USA (73); Austria, Tunisia and Russia (5); and Sweden, Norway, Finland, Denmark and Germany (32).

This evidence gap map is comprised of data from 3,136 participants. Sample sizes ranged from 10 to 567 with a mean age of participants ranging from 2.25 to 51.2 years. The ages reported in the studies ranged from 0.4 to 79 years. Out of the 58 studies, 35 of the studies focused on children and adolescents, with 23 reporting on interventions and outcomes across children, adolescents and adult age groups (see Appendix C). Seven studies did not report on the sex of participants [27, 30, 33, 38, 40, 48, 57] and in a further study [59] the sex of the participants was unclear. Out of the 51 studies that reported sex of the participants, there were 1,070 females and 1,036 males. The included studies were published between 1990 [22] and 2019 [43].

### Types of treatment and skeletal dysplasia

**Fig. 2:**
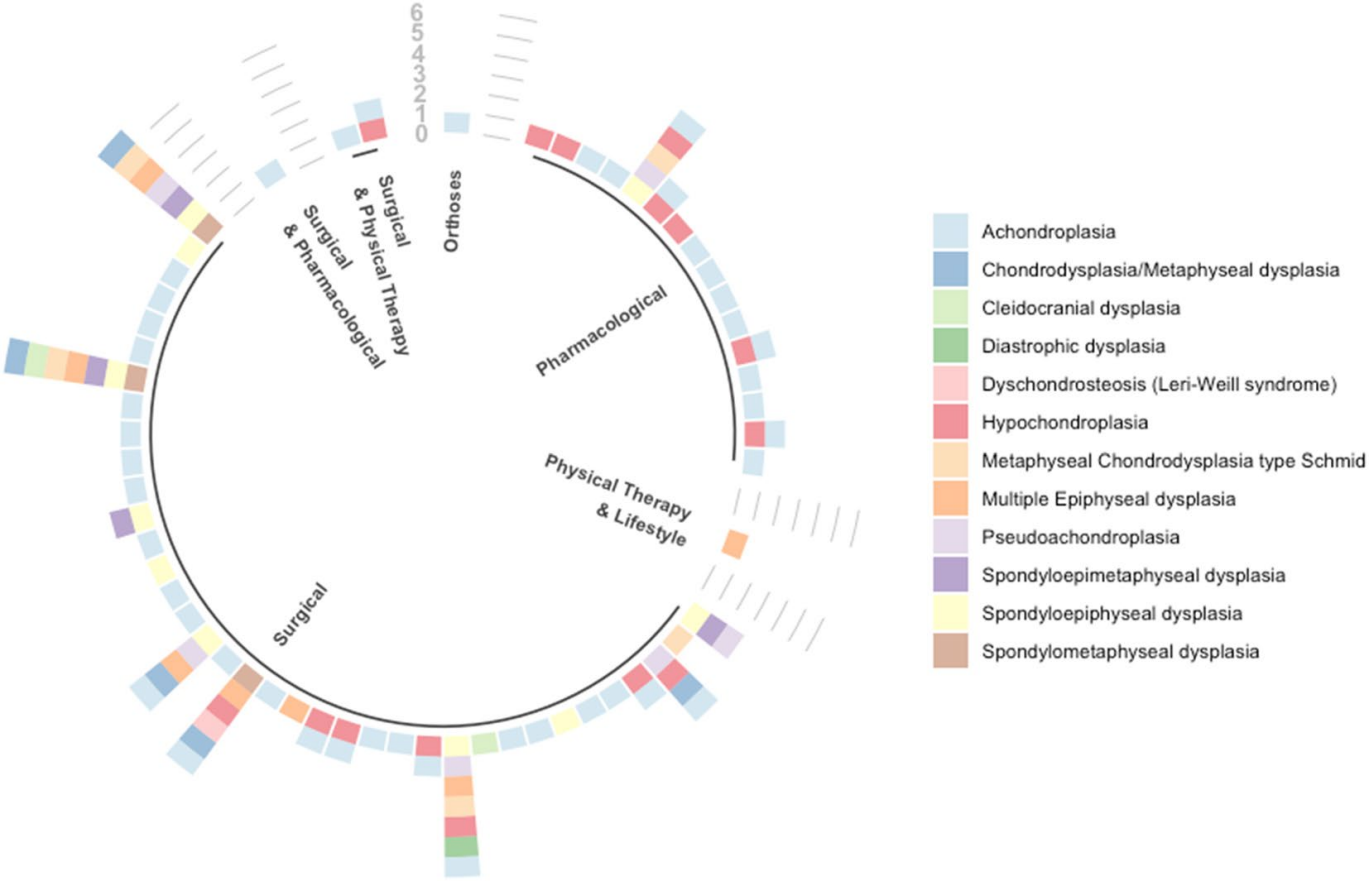
Evidence gap map—studies by type of skeletal dysplasia and treatment type

Included studies covered 12 types of skeletal dysplasia (see Fig. 2). 17 were multi-condition studies (including two or more skeletal dysplasias) [15, 19–21, 33, 34, 38–40, 45, 47, 56, 58, 64, 66, 73, 74]. Most studies focused on, or included people with Achondroplasia (ACH; *n* = 44), followed by Hypochondroplasia (HCH; *n* = 15), spondyloepiphyseal dysplasia (SED; *n* = 10), multiple epiphyseal dysplasia (MED; *n* = 7), pseudoachondroplasia (PAch; *n* = 6), chondrodysplasia/metaphyseal dysplasia (MD; *n* = 4), spondyloepimetaphyseal dysplasia (SEMD; *n* = 4), spondylometaphyseal dysplasia (SMD; *n* = 3), cleidocranial dysplasia (CCD; *n* = 2) and one study each for diastropic dysplasia and Leri Weill syndrome. Only five studies reported on treatments for individuals with MCDS [5, 15, 33, 66, 73].

The treatment focus of included studies across all skeletal dysplasias was primarily surgical (*n* = 40, 69% of studies), followed by pharmacology (*n* = 16, 28%), physical therapy and lifestyle behavioural change interventions (*n* = 1, 2%), and orthotic treatment (*n* = 1, 2%). Three of the surgical focused studies included additional treatments—one with pharmacological [43] and two with physical therapy [30, 74].

### Treatments and outcomes

**Fig. 3:**
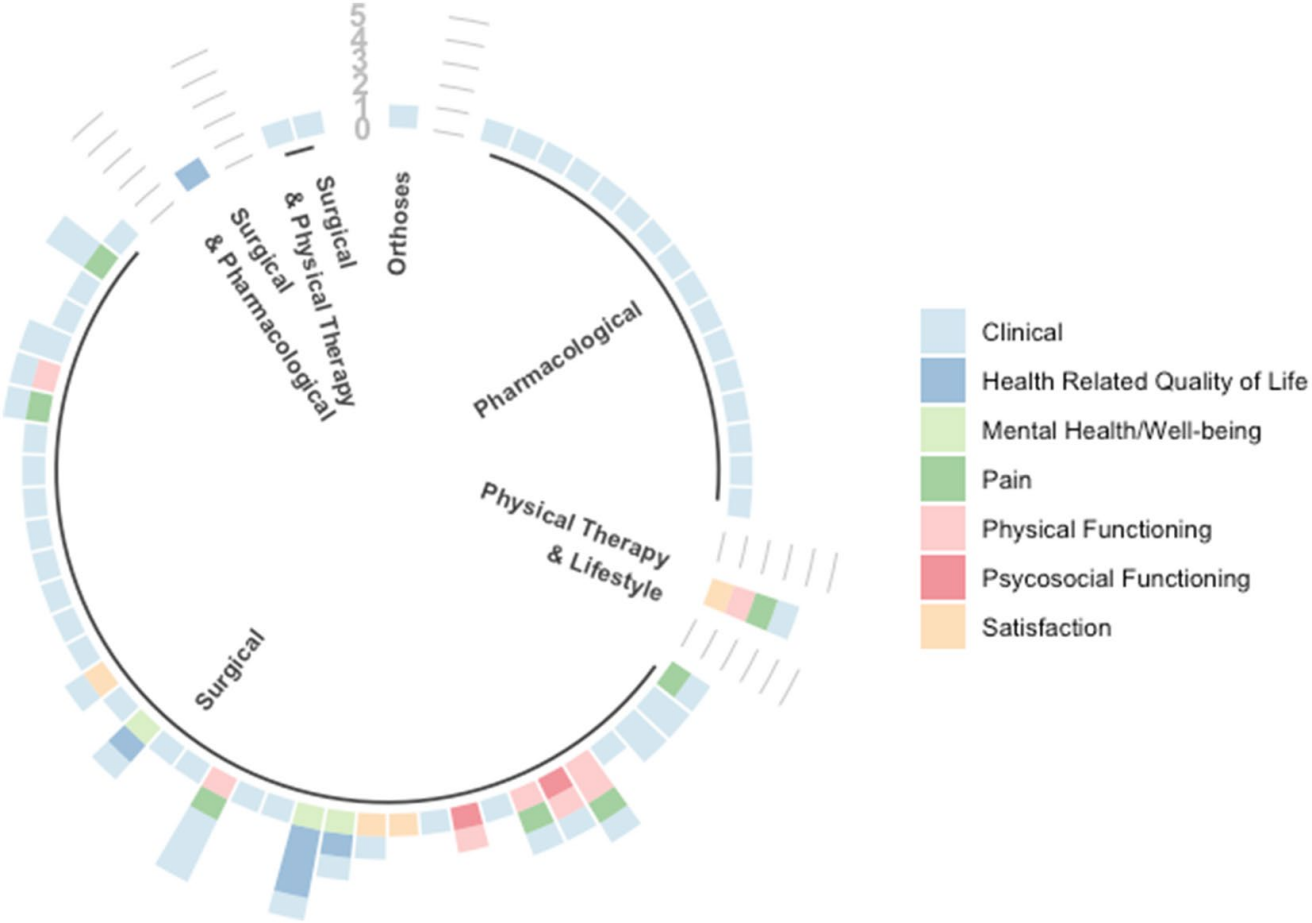
Evidence gap map—type of treatment and number of outcomes reported by study

Of the 40 studies of surgical treatment, 93% reported on clinical related outcomes such as increased length or height (see Fig. 3). Overall, out of the 58 included studies (*n* = 11, 19% reported on non-clinical outcomes (dimensions of HRQoL) such as their physical functioning and satisfaction with surgery. Two studies reported on use of physical therapy after surgical treatments for people with skeletal dysplasias [30, 45]. Despite this, physical functioning was assessed in only 10 studies, and was done primarily after surgery nine studies. One study that focused on individuals with MCDS reported on the effects of a pharmacological treatment on growth [15]. One study examined the effect of physical therapy after a lifestyle behaviour change intervention on physical functioning for individuals with MED, which was assessed using the Harris Hip Score (HHS) and a visual analogue scale (VAS) for pain and stiffness [37]. Only one study reported on use of orthoses that aimed to improve the extent of thoracolumbar kyphosis for children with achondroplasia [41]. Seven studies reporting on pain outcomes [23, 25, 28, 42, 70] after surgery assessed with symptom scores (HHS or VAS). One study reported on pain after physical therapy [45] and another after a lifestyle change intervention [37].

Dimensions of HRQoL was reported in four studies (6.8%) [35, 36, 43, 46]. Only one study reported on psychosocial functioning after surgery, while another reported on patient satisfaction with surgical treatment [24, 33]. Mental health/well-being outcomes were measured in three studies involving people with achondroplasia, which reported on changes in body image and self-esteem after surgery [35, 36, 46].

Of the three studies that reported physical therapy, none commented on specific outcomes linked to this treatment. Instead, these studies indicated that physical therapy was used as part of the treatment as a rehabilitative or clinical therapy combined with surgery [30, 45], or used in combination with other forms of conservative treatment [37].

### Surgery

Most of the evidence examining surgical treatments demonstrates potential improvement on clinical outcomes (e.g. growth—limb length and height, bone angle, hip alignment, dental outcomes) after surgical interventions; however, due to the variability of the conditions and the treatments it is not possible to compare these meaningfully. The effect of surgical interventions on HRQoL or dimensions of HRQoL were reported in studies involving Achondroplasia patients (see Table 1) [19, 23, 24, 28, 35, 36, 43, 46]. There seems to be no evidence of a difference on the HRQoL (assessed with SF-36) of surgical interventions of limb lengthening [35, 36, 46]. Additionally, there is no evidence of improvement in HRQOL (assessed with SF-36) for spinal surgery in people with achondroplasia [43]. There were no further studies reporting on the effect of surgical interventions using validated HRQoL instruments for any other type of skeletal dysplasia.

**Table 1:** Health-related quality of life measures and additional measures

Treatment type and condition	Health-related quality of life scale	Single item or other measures	Key finding
*Humeral lengthening (*monolateral external fixators): Achondroplasia [35]	SF-36 [75]: reports physical component summary (PCS), mental component summary (MCS), & total component summary (TCS)	Rosenberg self-esteem score [76]	Significantly improved the SF-36 score and Rosenberg self-esteem score
*Tibial and femoral lengthening*: Ilizarov ring fixator (Tibia), monolateral external fixators (Femoral): Achondroplasia [36]	SF-36 [75]: reports PCS, MCS & TCS PedsQL(for children between 8 and 12 years of age)[18]	Rosenberg self-esteem score [76]; AAOS lower limb outcomes [77]	Significantly improved the Rosenberg self-esteem score but no difference in other measures
*Humeral Lengthening* (external fixation with Ilizarov method): Achondroplasia [46]	SF-36 [75]: reports PCS & MCS	Rosenberg self-esteem score [76]	PCS, MCS and Rosenberg self-esteem score are only reported post-surgery. Scores are considered higher than those without lengthening surgery reported in the literature
*Multi-study examining historical treatment for height, humeral lengthening, orthodontics, andenotonsillectomy, ear ventilation tubing, foramen magnum surgery, spinal surgery:* Achondroplasia [43]	SF-36 [75]: reports PCS, MCS & role/social component summary	Self-reporting of getting in and out of vehicles, stepping up stairs, nursing care service use and wheelchair use after surgery	Treatment improved outcomes (single item and other measures) SF-36 scores significantly deteriorated after spinal surgery and showed no difference for the other treatments
*Spinal surgery* (lumbar decompression): Achondroplasia [23]	–	Symptom score: leg weakness, numbness or pain; incontinence; abnormal reflexes; walking tolerance	Treatment significantly improved outcomes
*Humeral lengthening* (monorail external fixators): Achondroplasia [24]	–	DASH score: disabilities to the arm, shoulder and hand	Treatment significantly improved outcomes
*Hip surgery* (osteotomy): SED Congenita [25]	–	Gait analysis and presence of hip pain	No difference in gait outcomes, some reduction in pain
*Spinal surgery* (historical laminectomy): Achondroplasia [28]	–	Independence indicated by RANKIN level [78]	No difference in independence level, however walking distance increased
*Hip conservative treatment (*limited weight bearing, control body eight, physical therapy and intermittent pain medications): MED [37]	–	Harris Hip Score [79]; visual analogue scale for stiffness [80]	Significantly improved outcomes in the Harris Hip Score and the visual analogue scale for stiffness
*Hip surgery (*total hip arthroplasty): MED [42]	–	Harris Hip Score	Significantly improved Harris Hip Score
*Hip surgery* (total hip arthroplasty): SED [71]	–	Harris Hip Score; Pain symptom score	Significantly improved Harris Hip Score and pain symptom score

In the study, examining historical interventions on HRQoL of people with Achondroplasia, the impact of foramen magnum surgery reported a mixed effect on HRQoL assessed with the SF-36 [43]. The findings were represented as a combination of the physical component summary (PCS), mental component summary (MCS), role/social component summary (RCS). Foramen magnum surgery significantly increased the MCS score, yet significantly reduced the RCS score; increased height also significantly increased the PCS score on the SF-36 [43].

Kim et al. (2012) reported no evidence of a difference in the HRQoL measures assessed with AAOS [77], total SF-36 [75] and PedsQl [81] after the intervention; however, a significant positive impact was observed in the Rosenberg self-esteem score with an absolute difference of 3.1 (*p* < 0.001) between the surgical and non-surgical group. Additionally, for the PCS of the SF-36, it provides weak evidence for lower HRQoL of patients who had surgery, with an absolute difference of − 10.59 (surgical score: 45.04; non-surgical score: 55.63; *p* = 0.0508).

A further study did not link HRQoL to surgical outcomes but did comment on the mental and psychological well-being of patients before surgery and the effect of the interventions on satisfaction after surgery [21]. In their study, Aldegheri and Dall’Oca [21] indicated that short stature had psychosocial implications that negatively impacted on their social and emotional relationships, although after surgery 95% would repeat the surgical procedure.

Pain appeared to improve with spinal surgery and hip alignment for those with PAch and SED, respectively [19, 25, 71]. For people with SEMD, hip alignment was not associated with any differences in pain, whereas an improvement in pain after surgery was not observed for people with MED [42, 66].

Physical functioning was improved in people with achondroplasia and MED after limb lengthening and hip alignment, respectively [24, 37, 42, 43]. There was an overall deteriorating effect of reducing independence after spinal surgery, as measured by a Rankin score in people with achondroplasia; improvements in independence were recorded by a point improvement of the Rankin score for those who receive surgery quickly (within 6 months of occurrence) [28].

One study examined individuals’ satisfaction after surgical intervention and observed that satisfaction is improved in hip alignment surgery and limb lengthening for SED, DD, PAch and MED, and in spinal surgery for DD [33]. However, lengthening deteriorated satisfaction for those with PAch [33]. A study on MED indicated no clinical complaints from patients were observed after surgery, supporting the findings for improvements for MED [37].

There was no evidence linking pharmacological treatments with HRQoL outcomes.

### Physical therapy

There were gaps in the evidence for the impact of physical therapy on clinical outcomes apart from weight bearing as measured via the HHS for people with MED [37]. Additionally there was no evidence for the impact of physical therapy on HRQoL or mental health/well-being.

### Lifestyle change and orthotic treatment

Gaps in the evidence were reported for the impact of health and lifestyle change interventions and orthoses on any outcomes, including HRQoL, with exception of spinal orthosis for improving height for people with achondroplasia [41] and the use of conservative treatment improving the HHS in people with MED [37].

## Discussion

This evidence gap map summarises the available treatment options and their impact on individual’s symptoms for individuals with non-lethal skeletal dysplasia with severe limb deformities and significant pain and discomfort that could lead to loss of mobility and require numerous orthopaedic interventions. Despite there being a number of studies available for the treatment of skeletal dysplasias, only five examine outcomes for people living with MCDS, with most articles focusing on Achondroplasia. Many of the treatment options available focus on clinical outcomes, with minimal investigation of their impact on HRQoL. Across all studies there is a focus on surgical and pharmacological interventions, with relatively few focussed on health and lifestyle modification, physical therapy, and orthotic treatment. Within the identified studies, the evidence suggested, overall, that surgical interventions may be likely to improve clinical outcomes, except for surgery for SMD and SED (hip alignment), SEMD (limb lengthening), and achondroplasia (spinal surgery). In studies of achondroplasia, the impact of treatment on HRQoL-related outcomes such as physical and psychosocial functioning, and mental health/well-being were prominent.

The inclusion of HRQoL outcomes after surgery was limited. Seven out of 58 publications discussed or briefly noted, the psychosocial and physical outcomes, and all were linked to surgical interventions, with the majority suggesting an improvement on activities of daily living, physical functioning, and presence of pain. However, some evidence indicates that certain surgical treatments do not lead to an improvement of psychosocial functioning. No evidence of a difference was found, however, for increased independence (psychosocial functioning) [28] after a laminectomy, the presence of stiffness did not show an improvement after conservative treatment [37] and gait showed no evidence of a difference post-operatively after a hip osteotomy [25]. A study reported reduced waddle after surgery in those with achondroplasia and HCH [74]. Patient satisfaction and, in some cases, parental satisfaction, was reported to be higher after some surgical procedures [33, 34, 37, 48].

The evidence gap map demonstrates that there are several gaps in evidence on non-surgical treatment and assessment of HRQoL for people living with skeletal dysplasias that were the focus of this study. We identified only four studies that explicitly examined the effects of treatments using validated HRQoL instruments with people living with skeletal dysplasia, and all used the SF-36 [75, 82] measure, further only one of these studies used the AAOS and PedsQl in conjunction with SF-36 [43]. Only one publication reported a significant improvement in HRQoL assessed with the SF-36 measure after surgical treatment with monolateral external fixators [35]. In a different study, spinal surgery significantly deteriorated SF-36 score [43]. It is evident that a gap exists on the effects of interventions on HRQoL in relation to SF-36 measurements, however this gap could be the result of the SF-36 score being validated for adults and not younger age groups. In addition, a further study [33] reported an apparently positive effect on satisfaction with surgery; however, there was no evidence that surgery for MCDS and PAch improved satisfaction with outcomes.

Despite identifying evidence that pharmacological and surgical treatments had positive effects on clinical outcomes for the dysplasias studied, these findings may not be generalised to types of skeletal dysplasia that were not eligible for inclusion in our study. For instance, the height results from growth hormone used for people with Achondroplasia may not be the same for people with CCD. In addition, due to the small number of publications on the use of physical therapy, health and lifestyle changes and orthoses [37, 41], more evidence is required. Further research should examine the preferences and experiences of individuals with skeletal dysplasias for treatments of symptoms and whether surgical and non-surgical treatments provide evidence of improved HRQoL. This research should also determine if there are age-specific differences linked to outcomes of HRQoL, as existing literature on HRQoL of demonstrates age and gender related differences [83].

To the best of our knowledge, this is the first evidence map on the outcomes of the treatments of non-lethal skeletal dysplasias. The strengths of this study is the methodological rigour with which it was performed, which included an extensive search strategy, a comprehensive summary of the results and an independent assessment on each stage of the study selection process. However, several articles were excluded for having fewer than 10 participants, which may have resulted in studies exploring the impact of treatment on a broader range of outcomes being missed. Additionally, due to the small sample sizes, the rarity of skeletal dysplasias in the general population, and the methodological variation amongst studies, the methodological quality of the studies included in the evidence-gap map could not be adequately assessed.

To conclude, there is a dearth of studies linking treatment of skeletal dysplasias with HRQoL outcomes. More research is needed to understand the lived experiences of those living with skeletal dysplasia, and how different treatment options impact on their HRQoL to inform decision-making about optimal treatment.

## Supplementary Information

Below is the link to the electronic supplementary material.

Supplementary file1 (DOCX 17 KB)

Supplementary file2 (DOCX 16 KB)

Supplementary file3 (DOCX 39 KB)

## Data Availability

Not applicable.

## Abbreviations

MCDS: Metaphyseal chondrodysplasia type Schmid
USA: United States of America
UK: United Kingdom
HRQOL: Health Related Quality-of-Life
ACH: Achondroplasia
CCD: Cleidocranial dysplasia
DD: Diastropic dysplasia
HCH: Hypochondroplasia
LWS: Leri Weillis syndrome
MD: Metaphyseal dysplasia
MED: Multiple epiphyseal dysplasia
PAch: Pseudoachondroplasia
SED: Spondyloepiphyseal dysplasia
SEMD: Spondyloepimetaphyseal dysplasia
SMD: Spondylometaphyseal dysplasia
