# Supplementary material for: Interventions for improving clinical outcomes and health-related quality-of-life for people living with skeletal dysplasias: an evidence gap map
Source: Qual Life Res. 2023 Jun 9;32(10):2751–62. doi: 10.1007/s11136-023-03431-z (PMC10474209; doi:10.1007/s11136-023-03431-z)
Supplement: Supplementary file 1 — Supplementary file1 (DOCX 17 KB) [file 11136_2023_3431_MOESM1_ESM.docx]

**Appendix A - Study Selection Form**

**Date: Researcher Initials: Study ID:**

| **Participants** | **Tick if present** |
| --- | --- |
| 1. Children or Adults with a diagnosis of |  |
| 1. Achondroplasia 2. Acromelic dysplasia 3. Acromesomelic dysplasia 4. Campomelic dysplasia 5. Cartilage‐hair hypoplasia (CHH) 6. Cleidocranial dysplasia 7. Diastrophic dysplasia 8. Dyschondrosteosis (Leri-Weill syndrome) 9. Eiken dysplasia 10. Epiphyseal dysplasia, multiple 2 11. Epiphyseal dysplasia, multiple 3 12. Epiphyseal dysplasia, multiple 5 13. Hypochondroplasia 14. Kniest dysplasia 15. Mesomelic dysplasia 16. Metaphyseal anadysplasia type 1 17. Metaphyseal anadysplasia type 2 18. Metaphyseal dysplasia, CHH‐like, POP1 type 19. Metaphyseal dysplasia, Jansen type 20. Metaphyseal dysplasia, McKusick type 21. Metaphyseal dysplasia, Schmid type (MCDS) 22. Metaphyseal dysplasia, Spahr type 23. Metaphyseal dysplasia without hypotrichosis 24. Metaphyseal dysplasia with maxillary hypoplasia 25. Metaphyseal dysplasia with pancreatic insufficiency and cyclic neutropenia (Shwachman Bodian Diamond syndrome) 26. Pseudoachondroplasia 27. Spondylometaphyseal dysplasia, Algerian type 28. Spondyloepiphyseal dysplasia congenita 29. Spondylometaphyseal dysplasia, Japanese type 30. Spondyloepimetaphyseal dysplasia, Strudwick type |  |
| **Interventions (at least one of the following)** |  |
| 1. Surgical interventions |  |
| 1. Physical therapy / physiotherapy |  |
| 1. Orthoses - externally applied device used to modify the structural and functional characteristics of the neuromuscular and skeletal system |  |
| 1. Pharmacological treatment (e.g analgesics for pain or growth hormone) |  |
| 1. Non-pharmacological treatment for pain (e.g heat pack) |  |
| 1. Supported self-management for health and lifestyle behaviour change |  |
| 1. Psychological therapies (e.g CBT), including counselling |  |
| 1. Combination treatment (mixture of the above) |  |
| **Outcomes (at least one of the following)** |  |
| 1. Clinical outcomes (e.g. limb alignment, posture, height, pain) |  |
| 1. Health-related quality of life or quality of life |  |
| 1. Mental health/well-being or resilience |  |
| 1. Psychosocial / psychological functioning, body image, self-esteem / concept |  |
| 1. Physical functioning |  |
| **Design** |  |
| 1. Quantitative or qualitative (or mixed methods) research studies with an abstract published in English from any country |  |

**IF 1 AND (2 OR 9) AND (10 OR 14) AND 15 are present 🡪 then INCLUDE**

**Include in review:** Yes/No/Unsure
