# Supplementary material for: Interventions for improving clinical outcomes and health-related quality-of-life for people living with skeletal dysplasias: an evidence gap map
Source: Qual Life Res. 2023 Jun 9;32(10):2751–62. doi: 10.1007/s11136-023-03431-z (PMC10474209; doi:10.1007/s11136-023-03431-z)
Supplement: Supplementary file 2 — Supplementary file2 (DOCX 16 KB) [file 11136_2023_3431_MOESM2_ESM.docx]

**Appendix B – Search Strategy (Embase)**

1 chondrodysplasia/

2 "metaphyseal chondrodysplasia type schmid".ti,ab,kw.

3 schmid metaphyseal chondrodysplasia.ti,ab,kw.

4 "metaphyseal chondrodysplasia schmid type".ti,ab,kw.

5 (metaphyseal adj (chondrodyplasia or anadysplasia or dyplasia or dysostosis)).ti,ab,kw.

6 schmid metaphyseal dysostosis.ti,ab,kw.

7 (collagen type x or COL10A1).ti,ab,kw.

8 collagen type 10/

9 ((type X or type 10) adj1 collagen).ti,ab,kw.

10 (Col X or Col 10).ti,ab,kw.

11 campomelic dysplasia/

12 growth disorder/

13 (skeletal dysplasia* or achondroplasia* or hypochondroplasia* or spondyloepimetaphyseal dysplasia* or eiken dysplasia* or cleidocranial dysplasia* or diastrophic dysplasia* or spondyloepiphyseal dysplasia* or kniest dysplasia* or spondylometaphyseal dysplasia* or epiphyseal dysplasia* or pseudoachondroplasia* or acromelic dysplasia* or acromesomelic dysplasia* or mesomelic dysplasia* or dyschondrosteosis or cartilage hair hypoplasia*).ti,ab,kw.

14 POP1 type.ti,ab,kw.

15 Jansen type.ti,ab,kw.

16 Spahr type.ti,ab,kw.

17 "Shwachman Bodian Diamond Syndrome".ti,ab,kw.

18 (disease* adj1 bone development*).ti,ab,kw.

19 (dwarf* adj1 short limb*).ti,ab,kw.

20 (genu varum or coxa vara).ti,ab,kw.

21 bone dysplasia/

22 dwarfism/

23 chondropathy/

24 (cartilage adj2 disease*).ti,ab,kw.

25 or/1-24

26 exp osteotomy/

27 orthopedic surgery/

28 exp computer assisted surgery/

29 surgical stapling/

30 osteotom*.ti,ab,kw.

31 (limb* length* and surg*).ti,ab,kw.

32 hemiepiphysiodesis.ti,ab,kw.

33 (surg* adj1 guided growth).ti,ab,kw.

34 (eight plate or 8 plate).ti,ab,kw.

35 (surg* adj1 (orthop?edic or treatment* or procedure* or operative)).ti,ab,kw.

36 physiotherapy/

37 (physical therap* or physiotherap*).ti,ab,kw.

38 kinesiotherapy/

39 exp exercise/

40 exercise*.ti,ab,kw.

41 exp drug therapy/

42 medication therapy management/

43 exp analgesic agent/

44 (pharmacolog* or medicine* or medication*).ti,ab,kw.

45 (drug adj (treatment* or therap*)).ti,ab,kw.

46 analgesic*.ti,ab,kw.

47 exp orthosis/

48 (orthotic* or orthos* or brace*).ti,ab,kw.

49 (support adj1 arch*).ti,ab,kw.

50 exp counseling/

51 (counseling or psycholog* or therap*).ti,ab,kw.

52 growth hormone/

53 (hormone adj1 (treatment* or therap*)).ti,ab,kw.

54 self care/

55 self manag*.ti,ab,kw.

56 (behavio?r* adj2 change*).ti,ab,kw.

57 ((hot or heat) adj2 pack*).ti,ab,kw.

58 or/26-57

59 abdominal pain/ or chronic pain/ or flank pain/ or exp musculoskeletal pain/ or neck pain/ or nociceptive pain/ or intractable pain/ or (pain adj1 (hip* or knee*)).ti,ab,kw.

60 referred pain/

61 (pain adj2 (manag* or reduc*)).ti,ab,kw.

62 convalescence/

63 functional residual capacity/

64 walking difficulty/

65 mobilit*.ti,ab,kw.

66 "growth, development and aging"/

67 growth/ or body growth/

68 fitness/

69 physical performance/

70 endurance/

71 body equilibrium/ or body position/

72 "quality of life"/ or quality adjusted life year/

73 (quality adj2 life).ti,ab,kw.

74 happiness/

75 child welfare/

76 child welfare/

77 mental health/

78 mental health.ti,ab,kw.

79 mental health recovery/

80 mental stress/

81 physical appearance/

82 body dysmorphic disorder/

83 psychological resilience/

84 coping behavior/

85 behavior/

86 health behavior/

87 child psychology/

88 wellbeing/

89 ((mental or psychological or emotional) adj (function* or wellbeing or well-being)).ti,ab,kw.

90 social isolation/

91 psychosocial care/

92 social class)

93 ((psychosocial or psycho-social) adj function*).ti,ab,kw.

94 exp academic achievement/

95 employment/ or employment status/

96 exp family relation/ or friendship/

97 leisure/

98 treatment outcome/

99 outcomes research/

100 childhood adversity/

101 risk assessment/

102 (treatment effect* or risk assessment* or adverse effect*).ti,ab,kw.

103 or/59-102

104 25 and 58 and 103

105 (animal/ or nonhuman/) not human/

106 104 not 105

107 limit 106 to (english or spanish or italian)
