# Supplementary material for: Interventions for improving clinical outcomes and health-related quality-of-life for people living with skeletal dysplasias: an evidence gap map
Source: Qual Life Res. 2023 Jun 9;32(10):2751–62. doi: 10.1007/s11136-023-03431-z (PMC10474209; doi:10.1007/s11136-023-03431-z)
Supplement: Supplementary file 3 — Supplementary file3 (DOCX 39 KB) [file 11136_2023_3431_MOESM3_ESM.docx]

**Appendix C - Summary of included study characteristics**

| **Author/Year** | **Country** | **Study Design** | **Conditions** | **Overall Mean Age** | **Type of Treatment** | **Treatments** | **Outcome(s)** |
| --- | --- | --- | --- | --- | --- | --- | --- |
| Ain et al. 2006 | USA | Retrospective prognostic study | SED, PAch, SEMD, Kniest, Morquio | 16.4 (*N* = 25) | Surgical | Posterior arthrodesis for the treatment of cervical instability | If union occurred, the neurologic outcome and complications of the intervention. |
| Al Kaissi et al. 2018 | Tunisia, Austria & Russia | Diagnostic & Clinical study | *MCDS* | - (*N* = 12) | Surgical | Bilateral proximal osteotomy to treat coxa vara; Bilateral valgisation osteotomy fixed with locking plate to treat radiographic appearance; Guided growth technique with hemiepiphyseodesis to treat valgus knee. | Radiographic imaging to demonstrate reduction in bowlegs. |
| Aldegheri 1999 | Italy | Retrospective cohort study | ACH, HCH, PAch, Chondropdysplasia, TS, ISS, Ellis-van Creveld, Rickets, Adrenogenital syndrome, Laron syndrome | 18.4 (*N* = 150) | Surgical | Distraction osteogenesis to lengthen bones. | Increased of the tibiae, osseous consolidation (healing index) and complications. |
| Aldegheri et al. 2001 | Italy | Retrospective cohort study | ACH, HCH, TS, ISS, Proportionate short stature, Dysmorphophobia | 16.4 (*N* = 140) | Surgical | Mid-shaft osteotomy with gradual distraction, Distraction of distal physis by means of chondrodiastasis and Distraction osteogenesis by callostasis to lengthen bones. | Increase in stature obtained; increase in length of the operated bone segments; complications and long-term sequelas; union time; total treatment time. |
| Appan et al. 1990 | UK | Case study, Retrospective diagnostic study and RCT | HCH | - (*N* = 84) | Pharmacological | Growth hormone therapy | Height difference (Standard deviation scores to mean height) |
| Baca et al. 2010 | USA | Retrospective case series | ACH | 10.6 (*N* = 18) | Surgical | Spinal decompression with or without instrumentation | Symptom score: differences between baseline, postop, revision postop and final follow-up. |
| Balci et al. 2015 | Turkey | Retrospective case series | ACH | 10 (*N* = 18) | Surgical | Monorail external fixators for bilateral humeral lengthening | Lengthening amount, bone function score (50), DASH score (Beaton et al., 12) |
| Bayhan et al. 2017 | USA | Retrospective case series | SED Congenita | 9.6 (*N* = 79) | Surgical | Valgus extension derotation osteotomy, with internal plate fixation | Hip pain, range of motion, presence of ossification, radiographic (Hilgenreiner trochanteric angle and neck shaft angle) and Sagittal Spinal Measurements |
| Beals et al. 2005 | USA | Retrospective cohort study | ACH | 8.5 (*N* = 22) | Surgical | Proximal tibial closing wedge osteotomy; Opening wedge tibial osteotomy of the tibia with external fixation with bicortical pins; tibia osteotomies below the tibia tubercle and distal fibular osteotomies with Ilizarov ring fixators | Angle correction of bowlegs, pre & post-op distance from the mechanical axis to the center of the knee joint. |
| Bridges et al. 1991 | UK | Case series | HCH | 9.6 (*N* = 31) | Pharmacological | Growth hormone therapy | Height standard deviation score (pre-treatment and up to 3 years post). |
| Carlisle et al. 2011 | USA | Retrospective analysis, survey | ACH | 37.7 (*N* = 49) | Surgical | Thoracolumbar laminectomy | Postoperative improvement in walking distance and independence level (Modified Rankin Scale level; (Sulter et al., 70)), intraoperative and post-op complications, and preoperative neurologic symptom severity. |
| Chang et al. 2015 | China | Retrospective cohort study | CCD | 28 (*N* = 15) | Surgical | Periodontal and endodontic treatments; Periodontal, endodontic and prosthodontic treatments; Comprehensive treatment, including periodontal, endodontic, surgical, orthodontic and prosthodontic treatments | Periodontal status, occlusion and aesthetics. |
| Donaldson et al. 2015 | UK | Retrospective case series | ACH | 7.8 (*N* = 10) | Surgical | Distraction osteogenesis with either an Ilizarov or Taylor spatial frame | Length gained and complications. |
| Harada et al. 2017 | Japan | Retrospective cohort study | ACH | - (*N* = 52) | Pharmacological | Growth hormone therapy with or without limb lengthening/gonadal suppression therapy | Height SD score at GH initiation (SD), for tibial and lengthening: standing height gain and theoretical lengthening value. |
| Hertel et al. 2005 | Denmark, Finland, Germany, Norway & Sweden | Clinical trial | ACH | 7.3  (Group 1)  7.1  (Group 2) (*N* = 35) | Pharmacological | Growth hormone therapy | Height velocity, height SD score, sitting height, arm span, sitting height ratios SDS, IGF-I SDS. |
| Hunter 1999 | Australia, Canada & UK | Survey | ACH, Cartilage-hair hypoplasia, Acromesomelia, Diastrophic dysplasia, HCH, Kniest, MED, PAch, *MCDS*, SED Congenita, Ellis van Creveld, SEDX-linked | ^ (*N* = 197) | Surgical | Understanding perceptions of surgical outcomes for: proximal femoral osteotomies, proximal tibial osteotomies, distal femoral osteotomies, amputations at the ankle, Achilles tendon lengthening, posteromedial release of feet, miscellaneous (Ilazarov for lengthening or joint alignment, stapling, bilateral hamstring releases, bilateral tibial distal osteotomies), foramen magnum-cervical fusions, scoliosis, thoracolumbar fusion/spinal decompression | Satisfaction score with surgery in terms of initial goals (disaster, much worse, bit worse, same, bit better, much better, completely better). |
| Kanazawa et al. 2003 | Japan | Clinical trial | ACH, HCH, PAch, *MCDS*, SED | ^ (*N* = 85) | Pharmacological | Growth hormone therapy | Height gain and height Z score, insulin-like growth factor. |
| Kashiwagi et al. 2001 | Japan | Retrospective case series | ACH, HCH | 12.8 (*N* = 10) | Surgical | Lengthening of the humeri using fixators (proximal or distal fixation) | Length gained, duration of external fixation, healing index, complications, some functional outcomes. |
| Kim et al. (a) 2012 | Korea | Retrospective case series | ACH | 11.8 (*N* = 12) | Surgical | Monolateral external fixators for lower limb lengthening *and QoL survey* | Humerus/femur lengthening, healing index, external fixator index, and complications. |
| Kim et al. (b) 2012 | Korea | Retrospective cohort study | ACH | 12.7 (*N* = 22) | Surgical | Ilizarov ring fixator for bilateral tibial lengthening; Monolateral external fixator for bilateral femoral lengthening; *and QoL survey* | Lengthening percentage, fixator index, bone healing index, complications and quality-of-life scores (AAOS lower limb outcomes; Rosenberg score; SF-36; Pediatric Quality-of-life). |
| Kim et al. 2017 | Korea | Retrospective prognostic study | MED | 9.6 (*N* = 40) | External | Conservative treatment: limited weight bearing with crutches or a cane, control of body weight, physical therapy to restore internal rotation and abduction, and intermittent pain medications. | Radiographic imaging of hips for center-edge angle, femoral head coverage, spherical index, acetabular depth index, acetabular angle, neck-shaft angle, Stulberg classification. Self-reported Harris hip score and visual analog scale score [pain and stiffness]. |
| Kitoh et al. 2007 | Japan | Retrospective cohort study | ACH, HCH | 16.3 (Treatment) 16.6  (Control)  (*N* = 20) | Surgical and clinical | Bone marrow cells and platelet-rich plasma on healing lengthened bones using monolateral external fixators. | Healing index and lengthening of femur/ tibiae. |
| Kitoh et al. 2014 | Japan | Retrospective case series | ACH, HCH | 14.6 (*N* = 24) | Surgical | Percutaneous osteotomy with monolateral external fixator and gradual distraction | Tibia/femur lengthening, healing index, fractures (complication), callus shape. |
| Kubota et al. 2016 | Japan | Longitudinal Cohort Study | ACH, HCH, ISS, GH deficiency, Short for gestational age | ^ (*N* = 58) | Pharmacological | Growth hormone therapy | Serum NT-proCNP levels, IGF-1, height SDS. |
| Leilei et al. 2018 | China | Retrospective case series | ACH | 2.3 (*N* = 39) | External | Brace | Thoracolumbar Kyphosis, lumbar lordosis, percentage of apical vertebra wedging for height, apical vertebral translation, pelvic tilt and pelvic incidence. |
| Lim et al. 2009 | Korea | Retrospective case series | MED | 47.2 (*N* = 14) | Surgical | Total hip arthroplasties | Center of rotation (horizontal, vertical), femoral offset, femoral neck length, limb length, complications. Additional radiographic measures.  Harris hip score, self-reporting of pain and functionality. |
| Matsushita et al. 2019 | Japan | Cross-sectional survey | ACH | - (*N* = 567) | Not specified | Investigating the impact of limb lengthening and hormone treatments on QoL | Health related quality-of-life (SF-36) for: physical Component Summary, mental component role/social component summary. |
| Mukherjee et al. 2014 | USA | Retrospective case series | ACH | 6.6 (*N* = 34) | Surgical | Cervicomedullary decompression | Postoperative polysomnography studies, changes in presenting signs and/or symptoms, and radiographic outcomes, such as change in ventricular size, presence or absence of CSF flow and diameter of the spinal cord in neutral, flexion and extension. |
| Myers et al. 2003 | UK | Retrospective case series | ACH, HCH, LWS, Chondrometaphyseal dysplasia, Osteochondromatosis, Olliers, SMD, Metaphyseal dysostosis, Ellis van Creveld, Fibrous dysplasia, Jeune syndrome, Melorheostosis, MED | 12.5 (*N* = 26) | Surgical | Distraction osteogenesis | Lengthening, angular correction, length and angular correction, reasons for failure (complications), healing index, lengthening index, and callus diameter ratio. |
| Nakano-Matsuoka et al. 2017 | Japan | Retrospective case series | ACH | 12.2 (*N* = 54) | Surgical | External fixation with Ilizarov method | Lengthening, healing index, external fixation period, distraction index, waiting period, width of osteotomy line and range of motion (elbow extension and elbow/shoulder flexion) and related complications. |
| Noonan et al. 1998 | Spain | Retrospective cohort study | ACH, SED, PAch, MED, ISS, Metaphyseal Chondrodysplasia, TS, Precocious puberty, Hypothyroidism, Pseudohypoparathyroidism, Kippel-Trénaunay syndrome, Congenital short femur, Congenital coxa vara, Hemihypertrophy, Post-traumatic growth arrest, Infectious growth arrest, Fibular hemimelia, Tibial hypoplasia, Ewing sarcoma, Poliomyelitis | 11  (Femoral)  13  (Tibia)  (*N* = 121) | Surgical | Distraction osteogenesis with monolateral external fixation | Lengthening, duration of distraction, healing indices, complications and additional revision surgeries (with the outcomes listed). |
| Park et al. 2003 | Korea | Retrospective case series | ACH | 15 (*N* = 10) | Surgical | Limb lengthening with a ring fixator | Lengthening index, complications, radiographic parameters (lumbar lordosis, lumbosacral joint angle, sacral inclinication angle, sacrohorizontal angle). |
| Park et al. 2015 | Korea | Retrospective case series | ACH | 10.8 (Femoral)  9.2  (Tibia)  (*N* = 28) | Surgical | Ilizarov ring fixator, monolateral external fixator for lengthening | Lengthening, lengthening index, healing index, external fixator index, serial pixel value ratios (at 28 weeks), and complications |
| Pinto et al. 2014 | France | Clinical Trial | HCH | 9.3 (*N* = 19) | Pharmacological | Growth hormone therapy | Standing height, sitting height and head circumference, sitting height-to-height ratio, BMI, GH/IGF-1 status, bone age, fat mass. |
| Ramaswami et al. 1999 | UK | Clinical Trial | ACH | 2.25 (*N* = 35) | Pharmacological | Growth hormone therapy | Height SDS, sitting height SDS, subsichial leg length SDS. |
| Savarirayan et al. 2019 | Australia, France, UK & USA, | Clinical Trial | ACH | 7.6 (*N* = 35) | Pharmacological | Vosoritide - an biologic analogue of C type natruiretic peptide drug | Adverse events, annualized growth velocity, height (mean Z score), Serum Biomarker Concentrations, estimated bone age/chronological age, upper-to-lower body segment ratio. |
| Seino et al. 1999 | Japan | Clinical Trial | ACH | 5.2  (Group 1)  6.2  (Group 2)  (*N* = 145) | Pharmacological | Growth hormone therapy | Height velocity (cm/year) and height velocity SDS. |
| Seino et al. 2000 | Japan | Clinical Trial | ACH | 5.2  (Group 1)  6.2  (Group 2)  (*N* = 145) | Pharmacological | Growth hormone therapy | Height velocity (cms) and height velocity SDS, height Z-score, growth rate, serum levels of IGF-I and IGFBP-3. |
| Serhan Er et al. 2017 | USA | Retrospective cohort study | SED Congenita | 6 (*N* = 20) | Surgical | Posterior cervical arthrodesis | Fusion achievement and decompression, immobilization period, revision surgeries, neural outcomes and complications. |
| Shohat et al. 1996 | USA | Clinical Study | ACH, HCH | 8.8 (*N* = 15) | Pharmacological | Growth hormone therapy | Height (SD), height velocity (cm/year), acceleration during hGH (cm/year), growth rates and long-bone growth rates, and biochemical levels. |
| Sisk et al. 1999 | USA | Retrospective case series | ACH | 3.8 (*N* = 36) | Surgical | Adenotonsillectomy, adenoidectomy or tonsillectomy | Effectiveness of adenotonsillectomy (changes in symptoms), revision surgery and complications. |
| Sitoula et al. 2014 | USA | Retrospective cohort study | SED, SEMD, Kniest, Morquio | 6.5 (*N* = 24) | Surgical | Occipitocervical fusion with new cable technique (Occiput to C2 fusion with or with-out C1 decompression) | Fusion, neurologic outcomes (mid-to-long term), and complications. |
| Song et al. 2018 | Korea | Retrospective case series | ACH | 11.1 (*N* = 34) | Surgical | Femoral lengthening using transverse osteotomy with a monolateral external fixator. | Range of movement (knee and hip), pelvic tilt, pelvic incidence, sacral slope and lumbar lordosis. |
| Song et al. (a) 2012 | Korea | Retrospective cohort study | ACH | 8.2 (Treatment) 8.5  (Control)  (*N* = 23) | Surgical | Bilateral tibial lengthening with monofocal proximal tibial osteotomy and the use of Ilizarov rings | Total length of the tibia, actual length of the tibia (without distraction), amount of physeal growth, growth rate and percentage growth. |
| Song et al. (b) 2012 | Korea | Retrospective cohort study | ACH | 8.2 (Treatment) 8.5  (Control)  (*N* = 35) | Surgical | Bilateral tibial lengthening by mono-focal proximal tibial osteotomy and the use of Ilizarov rights: with or without sequential femoral lengthening using mid-diaphyseal osteotomy and the use of a monolateral external fixator | Total length (mean total lengths of lower limbs), actual length of the lower limb, growth rate, percentage growth and physeal score. |
| Stamoyannou et al. 1997 | Greece | Clinical Study | ACH | 9.6 (*N* = 15) | Pharmacological | Growth hormone therapy | Height SDS, height velocity, sitting height and bone age to chronological age. |
| Tanaka et al. 1998 | Japan | Clinical Study | ACH | - (*N* = 42) | Pharmacological | Growth hormone therapy | Hypothalamic pituitary function (TSH response to TRH stimulus, and cortisol response to insulin-induced hypoglycemia), analysis of genomic DNA by restriction digest, mean Z-scores for height, height velocity, annual height gain, arm span to height ratio, lower limb length to height and sitting height. |
| Tanaka et al. 2003 | Japan | Clinical Study | ACH, HCH | 7.1  (ACH) 4.9 (HCH) (*N* = 15) | Pharmacological | Growth hormone therapy | Height (cm/year & SDS), height velocity (cm/year & SDS), bone age, bone mineral density (bone age & chronological age), IGF-I SDS. |
| Thomeer et al. 2002 | The Netherlands | Retrospective cohort study | ACH | 39.6 * (*N* = 36) | Surgical | Interapophyseolaminar Decompression | Level of decompression, symptom relief and complications. |
| Trigui et al. 2008 | France | Retrospective case series | SED Congenita, Kniest, MED, SEMD, Metaphyseal dysplasia, *MCDS*, CCD, SMD | 6.9 (*N* = 19) | Surgical | Femoral valgus, derotation osteotomy; Femoral valgus, extension, derotation osteotomy; Femoral valgus, extension osteotomy; Femoral valgus osteotomy; with or without Pelvic extension osteotomy | Hilgenreiner trochanteric angle, recurrence of coxa vara, symptoms of pain and stiffness. |
| Trivella et al. 1991 | Italy | Retrospective case series | ACH | 14.5 (*N* = 33) | Surgical | Chondrodiatasis (distraction of the epiphyseal plate) and callotasis (osteotomy) | Discusses: Sub-ischial leg length (SILL)/sitting height ratio, height, sitting height, and SILL, lower limbs (plantarflexion, inversion), lumbar lordosis and flexional mobility. |
| Vaidya et al. 2006 | Korea | Retrospective case series | ACH | 12.9 (*N* =24) | Surgical | Bifocal tibial osteotomy using Ilizarov ring fixator | Total tibial lengthening, lengthening at upper tibial osteotomy site, lengthening at lower tibial osteotomy site, healing index, complications, equinus contracture, medial proximal tibial angle, lateral distal tibial angle, mechanical axis deviation, lateral roximal femoral angle, mechanical lateral distal femoral angle, and joint line convergence angle at the knee joint. Tibiocalcaneal angle, posterior proximal tibial angle, anterior distal tibial angle, and posterior distal femoral angle, and tibial torsion. |
| Vleggeert-Lankamp et al. 2012 | The Netherlands | Retrospective case series | ACH | 51.2 (*N* = 20) | Surgical | Laminectomy and interapophyseolaminar decrompression | Symptoms (pre- & post-op), Cooper myelopathy score, mJOA Scale, Nurick Scale, Odom Scale, Thoracolumbar Kyphosis, interpedicular distance, anteroposterior distance. |
| Weiner et al. 2019 | USA | Retrospective cohort study | ACH | 6.1 (*N* = 27) | Surgical | Planned fibular nonunion | Anatomic tibio-femoral angle, tibia- fibula ratio, anatomic lateral distal femoral angle, lateral distal tibial angle, medial proximal tibial angle, mechanical axis zone, and tibia varus. Number of surgeries, surgical plumb-line category and complications. |
| Wyles et al. 2019 | USA | Retrospective case series | SED | 39 (*N* = 29) | Surgical | Total hip arthroplasty | Types of surgery, implant survival, revision surgery, pain, Harris Hip Score, and gait aids. |
| Yamate et al. 1993 | Japan | Clinical study | ACH | 6 (*N* = 22) | Pharmacological | Growth hormone therapy | Annual height gain, height velocity, growth curve, GH response to provocative tests, IGF-I level, months development of patients (head control, sitting down, walking, speech). |
| Yilmaz et al. 2014 | USA & Croatia | Retrospective case series | Morquio, MED, SED, SEMD, PAch, Kniest, Metaphyseal dysplasia, SMD, Ellis-van Creveld syndrome, *MCDS*, Stickler syndrome | 10 (*N* = 29) | Surgical | Guided growth: Temporary hemiepiphysiodesis using eight-plates and medial malleolus transphyseal screws. | Mechanical axis deviation, mechanical lateral distal femoral angle, lateral distal tibial angle, medial proximal tibial angle. |
| Zambito et al. 2000 | Italy | Retrospective cohort study | ACH, HCH, TS, ISS | ^ (*N* = 50) | Surgical and rehabilitative | Tenotomy of the Achilles tendon and plaster cast; Telescopic fixator, fasciotomy of the fascia lata, a tenotomy of the Achilles tendon and plaster cast and rehabilitative program | Range of movement, lengthening (cm, %), time of functional recovery, measurement of joint motion, muscle testing, analysis of gait. |

^ An overall age for the observations was not provided, instead the average age was provided for the respective condition groups.
* Average age at first surgery

*Notes*: ACH (achondroplasia), CCD (cleidocranial dysplasia), HCH (hypochondroplasia), ISS (idiopathic short stature), Kniest (Kniest syndrome), LWS (Leri Weill syndrome), MED (multiple epiphyseal dysplasia), Morquio (Morquio syndrome), PAch (pseudoachondroplasia), SED (spondyloepiphyseal dysplasia), SEMD (spondyloepimetaphyseal dysplasia), SMD (spondylometaphyseal dysplasia), TS (Turner syndrome)
